# Structure Learning in Bayesian Sensorimotor Integration

**DOI:** 10.1371/journal.pcbi.1004369

**Published:** 2015-08-25

**Authors:** Tim Genewein, Eduard Hez, Zeynab Razzaghpanah, Daniel A. Braun

**Affiliations:** 1 Max Planck Institute for Biological Cybernetics, Tübingen, Germany; 2 Max Planck Institute for Intelligent Systems, Tübingen, Germany; 3 Graduate Training Centre of Neuroscience, Tübingen, Germany; 4 University of Tübingen, Tübingen, Germany; University of Illinois at Chicago, UNITED STATES

## Abstract

Previous studies have shown that sensorimotor processing can often be described by Bayesian learning, in particular the integration of prior and feedback information depending on its degree of reliability. Here we test the hypothesis that the integration process itself can be tuned to the statistical structure of the environment. We exposed human participants to a reaching task in a three-dimensional virtual reality environment where we could displace the visual feedback of their hand position in a two dimensional plane. When introducing statistical structure between the two dimensions of the displacement, we found that over the course of several days participants adapted their feedback integration process in order to exploit this structure for performance improvement. In control experiments we found that this adaptation process critically depended on performance feedback and could not be induced by verbal instructions. Our results suggest that structural learning is an important meta-learning component of Bayesian sensorimotor integration.

## Author Summary

The human sensorimotor system has to process highly structured information that is affected by uncertainty and variability at all levels. Previously, it has been shown that sensorimotor processing is very efficient at extracting structure even in variable environments and it has also been shown how sensorimotor integration takes into account uncertainty when processing novel information. In particular, the latter integration process has been shown to be consistent with Bayesian theory. Here we show how the two processes of structure learning and sensorimotor integration work together in a single experiment. We find that when human participants learn a novel motor skill they not only successfully extract structural knowledge from variable data, but they also exploit this structural knowledge for near-optimal sensorimotor integration.

## Introduction

The sensorimotor system continuously integrates incoming information with previous experience across different modalities. Previous studies have shown that such integration processes in environments with uncertainty are consistent with Bayesian learning [1–6], where previous experience—the prior—and sensory evidence are weighted according to their reliability [7–12]. Several sensory illusions could be modeled and explained by the Bayesian integration of prior information with sensory feedback [13–18]. The same mathematical formalism also adequately describes integration of information from different sensory modalities, for example visual and haptic information [19, 20].

When integrating sensory information, it is important to know the statistics of the environment. Previous studies have mostly investigated Gaussian statistics factorizing into one-dimensional random variables, but a number of other distributions have been tested as well [8, 11, 19–25]. Here we are interested in the effect of the structure of the distribution given by the dependencies between multiple hidden variables that can be learnt as higher order invariants. Structural learning has previously been investigated in sensorimotor learning tasks with randomly changing task parameters [26–37]. In these previous studies it has been suggested that the sensorimotor system is faced with two concurrent learning problems in such randomly changing tasks, that is adapting to the current environmental parameters and extracting structural knowledge that remains invariant over many variations of environmental parameters.

In the current study we investigate the role of structural learning in a Bayesian sensorimotor integration task with a two-dimensional hidden variable that determined the two-dimensional displacement of visual feedback of the hand position. As in previous tasks, the value of the hidden variable has to be inferred during the integration process in each trial by combining sensory feedback with previous experience. Determining this value can be regarded as an example of parameter adaptation. However, since the hidden variable has two dimensions, we can also introduce structural dependencies between the two dimensions that remain invariant across trials. The question in the current study is whether and how such structural invariants of hidden variables influence the sensorimotor integration process of sensory feedback with prior experience.

## Results

### Trial setup

**Fig 1:**
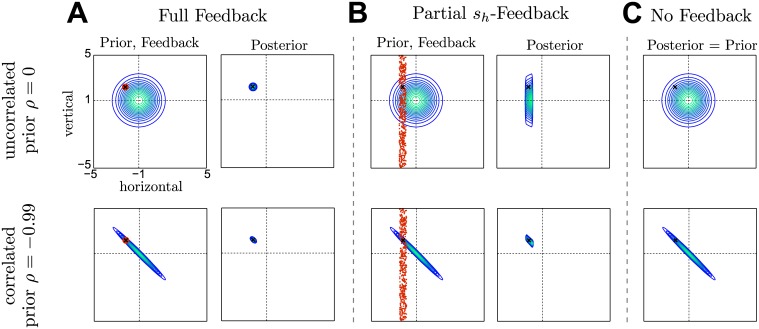
Bayesian integration for two-dimensional Gaussian priors under different feedback conditions **A**–**C**. The uncorrelated case is shown in the top row and the correlated case is shown in the bottom row. Prior and posterior are represented through iso-probability contours, the visual feedback is depicted in red and the true shift is marked as a black X. The black dotted lines indicate the prior mean. **A** Due to the very reliable feedback in the *full feedback* condition, the posterior is peaked very sharply—regardless of the correlation in the prior. **B** The *partial *s*_*h*_-feedback* is reliable in the *s*_*h*_ dimension but provides no information about the shift in the *s*_*v*_ dimension. This leads to an important difference in the posterior between the correlated and uncorrelated group: knowing the correlation structure reduces uncertainty about the *s*_*v*_ dimension of the shift, leading to a more concentrated posterior. **C** In *no-feedback* trials, participants can only rely on their prior experience. This feedback condition allows to test for the prior beliefs directly.

Participants performed a reaching task in a 3D virtual reality setup in which their virtual hand position was represented by a small spherical cursor. The aim of the task was to steer the cursor into a target sphere that was always at the same position in front of them. Similarly, the starting position was fixed throughout the experiment. To initiate a trial, participants had to move the cursor into the starting position. After a beep, the cursor disappeared and participants started their movement towards the target without visual feedback of their virtual hand position. Each trial, a two-dimensional translational shift was randomly drawn from a Gaussian, as depicted in Fig 1 and applied to the virtual hand position such that it was shifted with respect to the actual hand position. This shift was constant throughout the trial, but changed from trial to trial. Importantly, the Gaussian distribution over the shift remained constant over the course of the whole experiment.

Halfway through participants’ movement towards the target, feedback of the virtual hand position was briefly displayed for 150*ms*. This feedback was the only information participants could use to correct their shifted movement trajectory towards the target. There were four different feedback conditions that were chosen randomly in each trial with the following proportions: full feedback (1/2 of trials), partial *s*_*h*_-feedback (1/6 of trials), partial *s*_*v*_-feedback (1/6 of trials), and no-feedback (1/6 of trials). In the *full feedback* condition (Fig 1A) feedback was given by a small spherical cursor, which gave participants very precise information about the shift and allowed them to hit the target accurately. In the *partial *s*_*h*_-feedback* condition (Fig 1B) feedback was given by an elongated Gaussian cloud of points with width 0.6*cm* in the horizontal direction and full workspace width in the vertical direction. The cloud consisted of 50 small circles (radius 0.1*cm*) and their exact position was re-sampled several times during the display of the feedback, thus creating the visual effect of a flickering vertical bar, very similar to the depiction in Fig 1B. This sensory feedback gave relatively precise information about the horizontal shift *s*_*h*_, but no information about the vertical shift *s*_*v*_. In the *partial *s*_*v*_-feedback* condition this was reversed. The Gaussian cloud of points was elongated over the full workspace width in the horizontal direction and had a narrow vertical expansion of 0.6*cm*. Therefore, the sensory feedback provided relatively precise information about the vertical shift *s*_*v*_, but no information about the horizontal shift *s*_*h*_. In the *no-feedback* condition (Fig 1C) no feedback was provided, such that participants could only rely on their prior experience of the statistics in these trials. The critical feedback conditions are the partial feedback conditions. If the correlation structure between the two shifts is unknown, the feedback only provides information for one dimension. If, however, the correlation structure has been learnt over many trials, the partial feedback provides information for both dimensions of the shift.

### Sessions and groups

**Fig 2:**
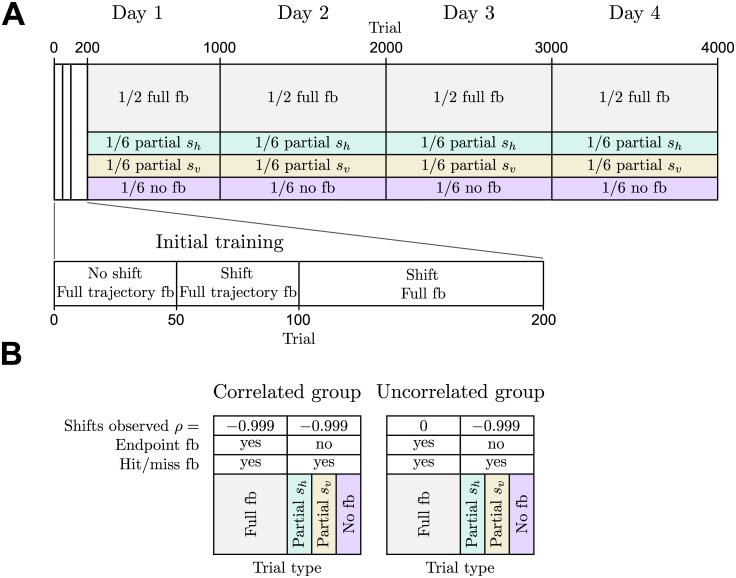
Schematic of the experimental design. **A** Participants were recorded over four sessions spread across four days. The first session included an additional training phase (first 200 trials) to allow participants to get used to the experimental setup. After this initial training phase, the different trial types were presented randomly according to the specified proportions. **B** Experimental conditions for the correlated and uncorrelated group.

Participants were recorded in this experiment over four different days—compare Fig 2A. The first six participants were assigned to the correlated group and the second six participants were assigned to the uncorrelated group. The correlated group was trained on shifts drawn from a correlated Gaussian (*ρ* = −0.999), while the uncorrelated group was trained on shifts drawn from an uncorrelated Gaussian (*ρ* = 0)—see Fig 2B. This training was given in full feedback trials, that not only provided most information during the movement compared to other feedback conditions, but also gave terminal feedback of the cursor position at the end of the trial. In contrast, all other feedback conditions served as test trials without terminal visuomotor feedback. However, to keep participants motivated they were informed in all feedback conditions whether they had hit the target or not through auditory feedback. To test whether participants of the correlated group were able to extract the structural invariant of the correlation in the hidden variable during training, in test trials (that is partial- and no-feedback trials) we exposed both groups to correlated shifts (*ρ* = −0.999) under partial- or no-feedback. In particular, we would expect the correlated group to differ from the uncorrelated group in the processing of the uninformative feedback dimension in partial feedback trials, as knowing the correlation structure allows transferring feedback information from the informative to the uninformative feedback dimension. In principle, the uncorrelated group could have also learnt the correlation in test trials by exploiting the hit-or-miss feedback provided in these trials. However, we would expect such reinforcement-learning to be much slower since the information of the reward feedback signal is poorer than the two-dimensional error signal observed by participants of the correlated group during training trials.

### Typical participant

**Fig 3:**
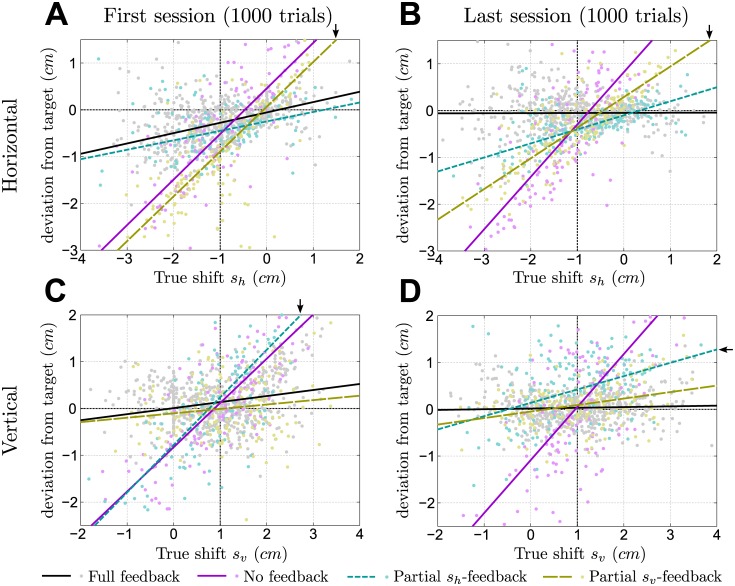
Data of a typical participant (no. 6, correlated group). The plots show the deviation of the final virtual hand position from the target as a function of the true shift. Dots represent individual trials and the lines show robust fits through the corresponding dots. Different colors indicate different feedback conditions. The crossing of the black dashed lines indicates the optimal pivot-point. Top row **A,B**: horizontal deviation as a function of the horizontal shift *s*_*h*_. Bottom row **C,D**: vertical deviation as a function of the vertical shift *s*_*v*_. The left column A,C shows results recorded in the first session of the experiment, the right column B,D shows results from the last session. In early trials, the participant’s reaction to partial feedback trials in the noninformative dimension is very similar to behavior in no-feedback trials. Importantly, across sessions there is a significant reduction in slope in the noninformative dimension of the partial feedback trials, indicating learning of the correlation structure (compare changes in lines highlighted with arrows, that is the yellow dashed lines in panels A and B and cyan dashed lines in panels C and D).

Fig 3 shows results of a typical participant from the correlated group performing in the task. The learning of the correlation structure can be seen when comparing the two panels on the left column showing performance in the first session to the two panels on the right column showing the last session. The plots show the deviation of the participant’s shifted terminal hand position from the target as a function of the true shift. The top row shows the horizontal deviation depending on the horizontal shift component *s*_*h*_, the bottom row shows the vertical deviation depending on the vertical shift *s*_*v*_. The different colors indicate the different feedback conditions. Each dot shows a single trial and the lines were robustly fitted through the corresponding dots (using MATLAB’s robustfit function with the default tuning constant of 4.685—the function implements iteratively re-weighted least squares with a bi-square weighting function). Flat lines indicate low performance error due to reliable feedback information. Lines with a unit slope indicate performance of a learner that exclusively relies on prior information. Lines with slopes in between these two extremes indicate a (Bayesian) weighting of feedback and prior information [8]. The mathematical predictions of the perfect Bayesian actor, that knows the statistics of the task and exactly compensates the mean of the posterior belief, can be seen for our task in Eq (2) of the methods. For this participant, in the full feedback condition (black lines) the slope was close to zero in both dimensions, as participants could see their virtual hand position relatively clearly and could therefore compensate the error regardless of the magnitude of the true shift. In contrast, in the no-feedback condition (purple lines) participants had to completely rely on their learnt prior and would ideally compensate the most probable shift, that is the mean shift. Accordingly, in no-feedback trials the participant’s deviation from the target as a function of the true shift is roughly described by a line with unit slope and intercept determined by the mean of the true shift—compare Fig 3.

### Analysis of partial feedback trials in the last session

If the participant had not learnt the correlation structure, performance measured by the slope in the partial feedback condition should be similar to the slope in the no-feedback condition with respect to the uninformative feedback dimension. This is exactly what we see in the early session depicted in Fig 3A and 3C showing that the mean of the distribution over shifts has been roughly learnt, but the correlation between the two dimensions of the shift has not been learnt. In contrast, we found a significant reduction in the slope of the uninformative feedback dimension after extensive training in the last session—compare yellow dashed lines in Fig 3A and 3B and cyan dashed lines in Fig 3C and 3D. This indicates that the correlation structure has been learnt partially over the course of 4,000 trials. If the correlation had been fully learnt we would expect all partial feedback lines in panels B,D to be very similar to the full feedback condition, that is having a slope close to zero.

**Fig 4:**
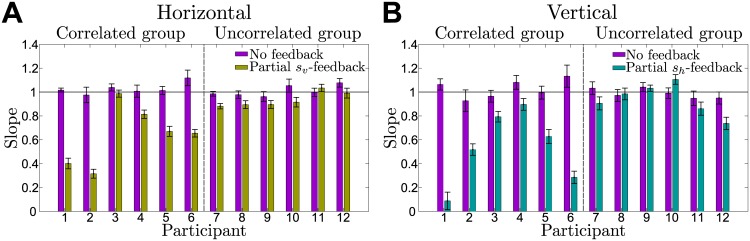
Performance of all participants in the last session of the experiment. The performance in the horizontal dimension is shown in panel **A**, performance in the vertical dimension in panel **B**. Performance is measured by slopes as in Fig 3 comparing no-feedback trials (purple) and partial feedback trials (yellow and cyan). Learning of the correlation structure is evident whenever the slope in the uninformative dimension of the partial feedback trials is significantly smaller than the slope in no-feedback trials (see also Fig 3). The perfect Bayesian response for no-feedback trials is characterized by a slope of one indicated by the thin black line, the Bayes-optimal slope for partial feedback trials would be zero—assuming that the Bayesian actor perfectly knows the statistics of the task. In both panels, error bars show standard errors of the robust fit.

The results for all participants of the correlated and uncorrelated group are shown in Fig 4, where in the last session of the experiment the correlated group shows a significant difference in slope in the uninformative feedback dimension of partial feedback trials compared to their performance in no-feedback trials (*p* = 0.030 signed-rank test for the horizontal slope and *p* = 0.030 signed-rank test for the vertical slope). The mean slope for the correlated group across participants in the last session was 0.640 ± 0.100 for the horizontal slope and 0.534 ± 0.125 for the vertical slope (mean ± standard error of the mean), which corresponds to the proportion of the perturbation that participants were not able to compensate. This suggests that information from the reliable dimension in partial feedback trials was successfully applied to the dimension providing no feedback which is only possible if the correlation structure has been learnt at least partially. In contrast, the uncorrelated group did not show a significant difference in slope in the uninformative feedback dimension of partial feedback trials compared to their performance in no-feedback trials (*p* = 0.438 signed-rank test for the horizontal slope and *p* = 0.063 signed-rank test for the vertical slope). The mean slope across participants of the uncorrelated group in the last session was 0.935 ± 0.025 for the horizontal slope and 0.937 ± 0.054 for the vertical slope (mean ± standard error of the mean). In principle, however, this group could have adapted their slope through reinforcement learning in partial and no-feedback trials, which might explain the close-to-significant p-value in the vertical dimension. More importantly, therefore, comparing the reduction in slope from the no-feedback to the partial-feedback trials between the correlated and uncorrelated group, we find a significant difference between both groups (*p* = 0.041 rank-sum test for the horizontal dimension and *p* = 0.009 rank-sum test for the vertical dimension, data from last session).

### Analysis of no-feedback trials in the last session

**Fig 5:**
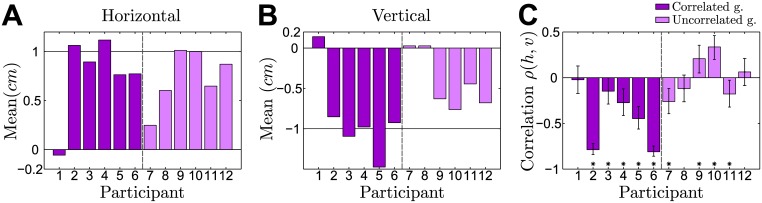
Means and correlation of the final hand position in no-feedback trials of the last session. **A** The mean of the final horizontal hand position for an ideal actor should be +1*cm* to fully compensate the mean shift. **B** The mean of the final vertical hand position for an ideal actor should be −1*cm* to fully compensate the mean shift. **C** Correlation coefficient between the vertical and horizontal components of the final hand position. Error bars indicate 95% confidence intervals—bars marked with a star show significant correlations (at a 5% level).

In no-feedback trials, participants can only rely on their experience from previous trials, which allows to directly query their prior belief about the expected shift by investigating participants final hand positions. Fig 5A and 5B shows the mean of participants’ final hand positions in no-feedback trials over the last session. To perfectly compensate the mean of the experimentally induced distribution over the shift, participants should on average reach to [1,−1]*cm* in order to maximize their hitting probability. This holds for both the correlated and the uncorrelated group, since the mode of the distribution over the shift is unaffected by the correlation. As shown in Fig 5A and 5B we found that most participants learnt the mean shift with no significant difference between the correlated and the uncorrelated group (*p* = 0.590 for the horizontal dimension and *p* = 0.065 for the vertical dimension, rank-sum test).

Moreover, we computed the correlation coefficient between the vertical and horizontal components of participants’ final hand position in no-feedback trials of the last session of the experiment. As shown in Fig 5C, we found a significant difference between both groups (*p* = 0.041, rank-sum test)—participants from the correlated group systematically showed a negative correlation in their final hand-position (*p* = 0.030, sign test), whereas participants from the uncorrelated group did not (*p* > 0.990, sign test). Importantly, a correlation in the two dimensions of the hand position cannot be explained by a perfect Bayesian actor model that exactly compensates the mean of the prior distribution over the shifts, even if some isotropic motor noise was added to this planned response. We consider two hypotheses that are not necessarily mutually exclusive. The first hypothesis is that the correlation could be a signature of a bounded rational actor that samples beliefs from its prior distribution over shifts and chooses its actions with regard to these samples. The second hypothesis is that the correlation simply reflects the correlation in the previous full feedback trial assuming a trial-by-trial adaptation process. We found evidence for both hypotheses. In particular, we found in accordance with the second hypothesis that participants’ responses in no-feedback trials of both the correlated and uncorrelated group were significantly correlated with the shift of the previous full feedback trial (correlated group last session: correlation-strength −0.31 ± 0.26 horizontal and −0.34 ± 0.23 vertical (mean ± standard deviation)—uncorrelated group last session: correlation-strength −0.2 ± 0.15 horizontal and −0.23 ± 0.13 vertical). For the correlated group this correlation was significant for four out of six participants in the horizontal dimension and for five out of six participants in the vertical dimension—for the uncorrelated group we found a significant correlation for four out of six participants in both dimensions. If the correlation in the two dimensions of the hand position was entirely due to trial-by-trial adaptation, we would expect the correlation to be roughly stationary, as the xy-correlation in full feedback trials is already present in the earliest trials of the first session and changes only minimally across sessions. In contrast, we found that the correlated group started with a close-to-zero xy-correlation in no-feedback trials and showed learning-dependent improvement in the correlation over time (xy-correlation coefficient across participants in the first session −0.12 ± 0.17 versus the last session −0.42 ± 0.33, mean ± standard deviation), which would fit with the predictions of a bounded rational model of acting—compare Section: Model prediction.

### Learning across sessions

**Fig 6:**
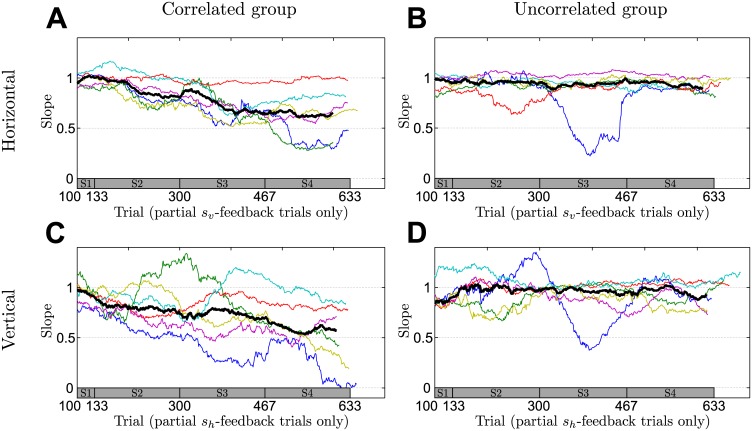
Changes in slope in partial feedback trials. The slope is a performance measure determined as in Fig 3 but using a sliding window of 100 trials. **A** Evolution of the horizontal slopes in partial *s*_*v*_ feedback trials of the correlated group where horizontal information is not given by the feedback, but can only be obtained through knowledge of the correlation structure. **B** Same as A but showing data of the uncorrelated group. **C** Evolution of the vertical slopes in *s*_*h*_ feedback trials of the correlated group where vertical information is not given by the feedback, but can only be obtained through knowledge of the correlation structure. **D** Same as C but showing data of the uncorrelated group. For the analysis only partial *s*_*v*_- or partial *s*_*h*_-feedback trials were taken out from the pooled data across all sessions. Thin colored lines indicate individual participants and can vary in length since the exact number of relevant trials could fluctuate due to the probabilistic generation of trials. The thick black line shows the median over participants—taking only into account trials where data from all participants exists. The bar at the bottom of the figure indicates the corresponding session (on average).

**Fig 7:**
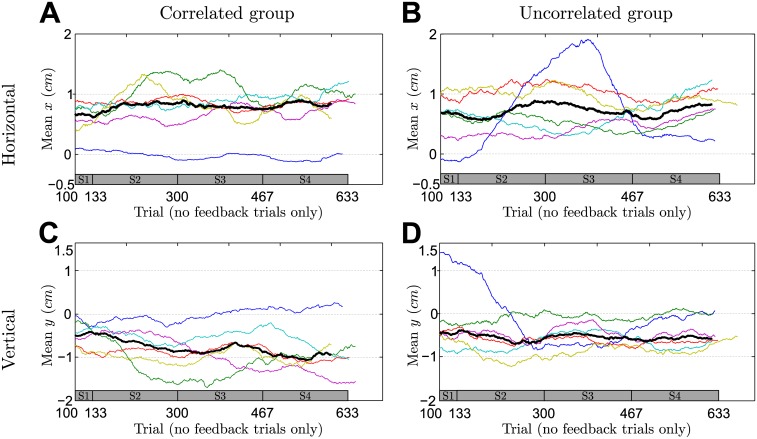
Learning of mean shift over all sessions revealed by performance in no-feedback trials averaged over a sliding window of 100 trials. **A** Learning of the mean in the horizontal dimension of the correlated group. **B** Same as A but showing data of the uncorrelated group. **C** Learning of the mean in the vertical dimension of the correlated group. **D** Same as C but showing data of the uncorrelated group. For the analysis only no-feedback trials were taken out from the pooled data across all sessions. Thin colored lines indicate individual participants and can vary in length since the exact number of relevant trials could fluctuate due to the probabilistic generation of trials. The thick black line shows the median over participants—taking only into account trials where data from all participants exists. The bar at the bottom of the figure indicates the corresponding session (on average).

To investigate behavior beyond the final session, we analyzed the dynamics of learning over the entire four sessions. We assess the evolution of participants’ performance slopes in partial feedback trials and in no-feedback trials the evolution of participants’ correlation between the two dimensions of their final hand position as well as the evolution of their mean responses. Fig 6 shows participants’ evolution of performance slopes across the four sessions in partial feedback trials. The figure shows individual participants as thin colored lines and the median over participants as a thick black line. The results show a clear difference between the correlated and the uncorrelated group—the correlated group shows a steady decrease in slopes across sessions, whereas the uncorrelated group shows no such trend. This suggests that the correlated group gradually learnt to harness the informative feedback dimension to facilitate the sensorimotor integration process in the uninformative feedback dimension. In contrast to the gradual learning of the correlation structure, we found no difference in learning of the mean of the distribution over shifts between the two groups—compare Fig 7. The results suggest that large parts of learning the mean shift already happened before the occurrence of the first no-feedback trials that we used to assess learning of the mean in the figure.

**Fig 8:**
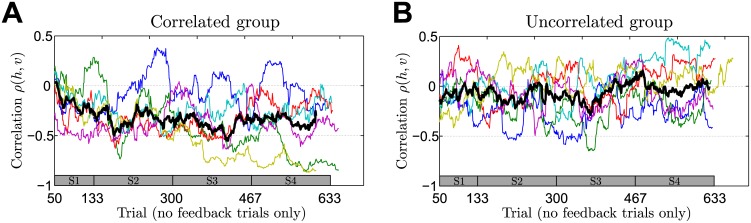
Adaptation of correlation between the vertical and horizontal terminal hand position measured in no-feedback trials. Correlation values are determined in a sliding window of 50 trials across all four sessions. **A** Correlated group. **B** Uncorrelated group. For the analysis only no-feedback trials were taken out from the pooled data across all sessions. Thin colored lines indicate individual participants and can vary in length since the exact number of relevant trials could fluctuate due to the probabilistic generation of trials. The thick black line shows the median over participants—taking only into account trials where data from all participants exists. The bar at the bottom of the figure indicates the corresponding session (on average).

Finally, we investigated the evolution of participants’ correlation between the two dimensions of their final hand position. In Fig 8A and 8B we show the evolution of the correlation coefficient between the horizontal and vertical component of participants’ final hand position in no-feedback trials over the course of the whole experiment. Similar to the results in Fig 5C, we found that the correlated group shows an increasingly negative correlation across sessions, whereas the uncorrelated group does not show such a trend.

### Control experiment: reinforcement learning vs. supervised learning

In our experimental design the correlated group could have learnt the correlation structure from two sources: first, from the error signal in full feedback trials allowing for some kind of supervised learning, and second, from the binary auditory performance feedback in partial and no-feedback trials allowing for some kind of reinforcement learning. As the uncorrelated group experienced the same statistics and binary performance feedback in partial and no-feedback trials, we can already exclude the possibility that the correlation structure in partial and no-feedback trials is learnt from binary feedback alone. However, it is unclear whether the binary feedback signal was crucial for the correlated group in learning the correlation structure.

**Fig 9:**
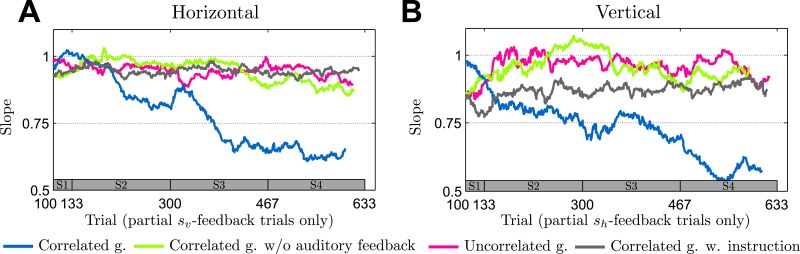
Changes in slope in partial feedback trials across groups. The slope is determined as in Fig 6. **A** Evolution of the horizontal slopes in partial *s*_*v*_ feedback trials where horizontal information is not given by the feedback, but can only be obtained through knowledge of the correlation structure. **B** Evolution of the vertical slopes in *s*_*h*_ feedback trials where vertical information is not given by the feedback, but can only be obtained through knowledge of the correlation structure. The correlated group shows a gradual and steady improvement across sessions whereas the other groups do not show such a trend. Different colored lines show the median over the different groups of participants and can vary in length since the exact number of relevant trials could fluctuate due to the probabilistic generation of trials. The bar at the bottom of the figure indicates the corresponding session (on average).

To control for this possible source of learning, we devised a control group that underwent the same experimental procedure as the correlated group with the important exception that this group did not receive any performance feedback in partial and no-feedback trials. We found that this group behaved similarly to the uncorrelated group in that they showed almost no reduction in slope in partial feedback trials (p = 0.485 horizontal and p = 0.699 vertical, ranksum test against uncorrelated group with data from the final session), in clear contrast to the correlated group that received binary performance feedback in partial and no-feedback trials (p = 0.041 horizontal and p = 0.026 vertical, ranksum test against correlated group with data from the final session). The same pattern is also visible in the evolution of slopes across sessions as shown in Fig 9 (evolution of slopes of individual participants is shown in Supplementary S1 Fig, evolution of means of individual participants is shown in Supplementary S2 Fig). This suggests that participants require both signals to learn, that is the immediate auditory feedback in partial- and no-feedback trials and the endpoint feedback reflecting the correlation structure in full-feedback trials.

### Control experiment: cognitive strategies vs. motor learning

In our experimental design the optimal strategies in full and partial feedback conditions of the correlated group always required diagonal compensatory movements that were either directed left-up or right-down. This raises the question of whether participants could have learnt an explicit cognitive strategy instead of implicit sensorimotor integration. The hypothesis is that an explicit cognitive strategy can be verbally communicated and enable the participant more or less instantly to perform well. To control for this possibility, we devised a group of participants that was explicitly informed about the correlation structure, that is they were told that successful compensations would either be left-up or right-down. Crucially, if the correlated group simply learnt a cognitive strategy then the explicitly instructed group should be able to perform in their first session as well as the correlated group in their last session, assuming that the correlated group had figured out the cognitive strategy by the fourth session that the instructed group was given immediately. We found this not to be the case.

In the partial feedback trials, the correlated group performed significantly better at the end of the experiment than the instructed group in their first session (comparing the reduction of slope in the first session of the instructed group against the reduction of slope in the last session of the correlated group with a ranksum test: p = 0.026 horizontal dimension in partial *s*_*v*_ trials and p = 0.065 vertical dimension in partial *s*_*h*_ trials). The performance difference between the two groups is particularly obvious when comparing the evolution of the slope in partial feedback trials across sessions—compare Fig 9. The figure shows that the instructed group is not learning the correlation structure across sessions, as there is no statistical evidence for improvements of the slopes in partial feedback trials across sessions (comparing the reduction in slope with respect to the no-feedback slope between the first and the last session of the instructed group with a ranksum test: p = 0.937 horizontal and p = 0.937 vertical). The evolution of slopes of individual participants is shown in Supplementary S1 Fig and the evolution of means of individual participants is shown in Supplementary S2 Fig. There is, however, evidence that participants understood the instructions, as they showed a significant correlation between the horizontal and vertical dimension of their hand movements under partial feedback straight away: in the first session, the instructed group had a movement-correlation in horizontal and vertical partial feedback trials of −0.29 ± 0.15 and −0.27 ± 0.07 (mean ± standard deviation) respectively compared to the correlated group that showed no initial correlation of the horizontal and vertical dimension of their hand-movement in partial feedback trials (−0.06 ± 0.16 and −0.04 ± 0.17, mean ± standard deviation). This difference in correlation was significant (p = 0.026 horizontal and p = 0.026 vertical, ranksum test comparing the first session of the correlated group and the first session of the instructed group). The evolution of the xy-correlation in partial feedback trials across all sessions draws the same picture—compare Supplementary S3 Fig.

Surprisingly, the increased correlation in the hand movements in partial feedback trials of the instructed group did not produce a reduction in slope in these trials. In fact, the instructed group showed strongly increased slopes in the low-uncertainty dimension of the partial feedback trials in the first session of the experiment—compare Supplementary S3 Fig. In the low-uncertainty dimension of the partial feedback trials, an ideal actor should have a slope close to zero reflecting low uncertainty about the shift. The instructed group had an elevated slope of 0.725 ± 0.469 and 0.732 ± 0.455 (mean ± standard deviation) for horizontal and vertical partial feedback trials in the first session respectively, compared to the correlated group that had a slope of 0.342 ± 0.160 and 0.193 ± 0.154 (mean ± standard deviation) in their first session. This suggests that, while the instructions were clearly understood and followed, the explicit instructions actually impeded participants’ ability to compensate the shifts in the low-uncertainty dimension of the partial feedback, particularly in the early sessions of the experiment. As performance in the low-uncertainty dimension does not require learning a statistical prior (and in fact in all the other groups there seems to be better performance and little performance improvement in the low uncertainty dimension—see Supplementary S3C and S3D Fig), this suggests that the deficient performance in the instructed group might be due to a shift in attentional focus, where subjects might pay more attention to following the instruction than to actual performance [38]. Further, the instructed group also shows impeded implicit learning—compare the evolution of the slope in partial feedback trials for the instructed group in Fig 9. While these results are not conclusive with respect to the origin of the deficient performance of the instructed group, they clearly demonstrate that explicit instructions did not instantly improve performance and therefore suggest that the correlated group were not following an explicit cognitive strategy.

### Model predictions

**Fig 10:**
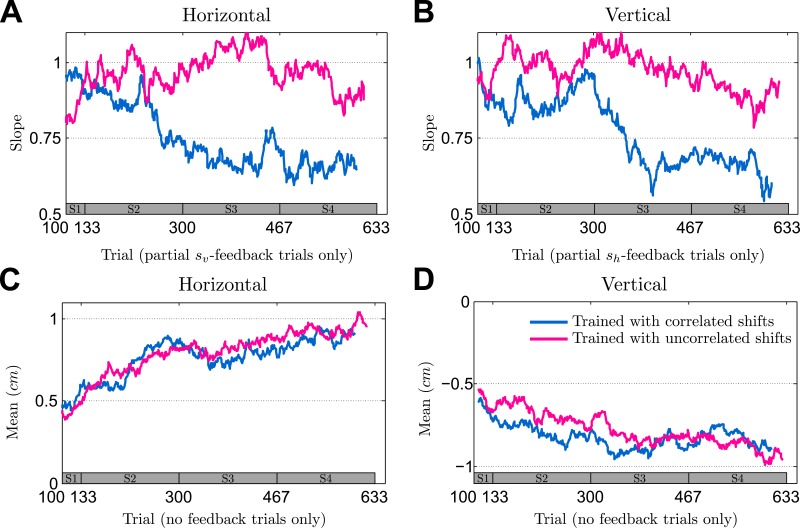
Simulation results. The plots show the simulation results as medians over six different simulation runs. Blue lines show the medians over six runs where the model was trained with correlated full feedback trials. Pink lines show the medians over six run where the model was trained with uncorrelated full feedback trials. **A** Median for evolution of horizontal slope in partial *s*_*v*_-feedback trials—compare Fig 9A which shows the participants’ results. **B** Median for evolution of vertical slope in partial *s*_*h*_-feedback trials—compare Fig 9B which shows the participants’ results. **C** Median for evolution of horizontal mean-response in no feedback trials—compare Fig 7 and Supplementary S2E Fig which shows the participants’ results. **D** Median for evolution of vertical mean-response in no feedback trials—compare Fig 7 and Supplementary S2F Fig which shows the participants’ results. The parameters of the model (strength of the initial belief over mean-shift and covariance matrix) were chosen in order to minimize the sum-of-squared-differences between the correlated simulation median and the median obtained from participants from the correlated group. The uncorrelated simulation run used the same set of parameters.

As in the model described in [8], the ideal Bayesian actor optimally integrates prior knowledge about the shift with feedback information in each trial. For our experiment this mathematical prediction can be found in Eq (2) of the Methods. Importantly, this integration presumes that the prior is perfectly learnt to be consistent with the experimentally imposed prior. While this is the case in [8], in our study this is not the case, as can be seen for example in Fig 9, where the slopes in partial feedback trials never approach the Bayesian optimum of zero. This implies that the correlation in the prior is never fully learnt by participants. To model participants’ behavior we therefore devised not only a Bayesian model of sensorimotor integration of prior and feedback, but also a Bayesian model of learning the prior and the corresponding correlation structure. In this model the actor has a belief about the prior over the shift *s* given by *p*(*s*∣*μ*_0_,Σ_0_), where *μ*_0_ and Σ_0_ are hyper-parameters that the actor is learning over the course of many trials. If the initial belief over *s* is concentrated on uncorrelated shifts, as would be plausible for everyday planar movements, the model can explain partial learning of the correlation structure—compare Fig 10.

**Fig 11:**
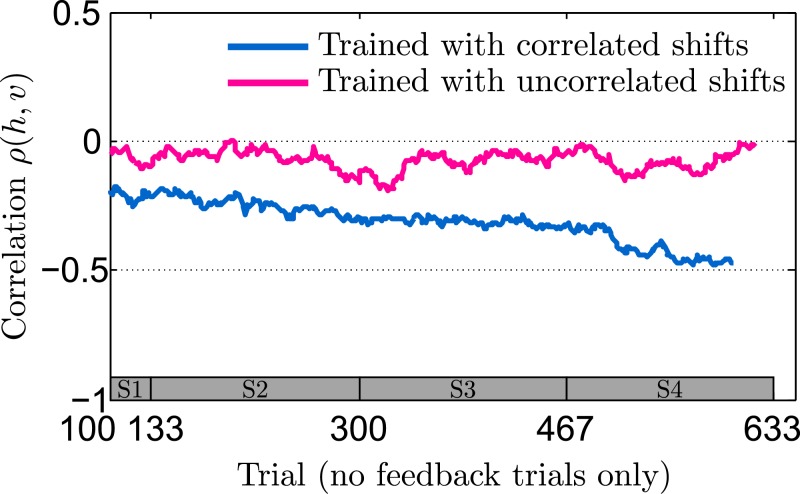
Simulation results. Median for evolution of xy-correlation in no feedback trials—compare Fig 8A and 8B which shows the participants’ results. The plot shows the simulation results as medians over six different simulation runs. The blue line shows the median over six runs where the model was trained with correlated full feedback trials. The pink line shows the median over six runs where the model was trained with uncorrelated full feedback trials. The parameters of the model (strength of the initial belief over mean-shift and covariance matrix) were chosen in order to minimize the sum-of-squared-differences between the correlated simulation median and the median obtained from participants from the correlated group. The uncorrelated simulation run used the same set of parameters.

Fig 10 shows the median of performance slopes and mean-responses under two different training conditions, each with six exemplary runs of the model. The blue curves show model predictions when trained on correlated trials, the pink curves show model predictions when trained on uncorrelated trials. Independent of the training regime the model predicts that both groups of participants should learn the mean shift equally well, which is in line with our experimental findings. In the case of partial feedback trials, the model predicts that the uncorrelated group shows no learning and should have a slope close to one. In contrast, when trained on correlated trials, the model predicts that the slope should decrease over time, indicating gradual learning of the correlation structure. Moreover, if actions are determined by samples from the distribution over shifts, the model predicts that the xy-correlation in no-feedback trials should gradually increase in magnitude when trained on correlated trials, whereas no such trend should be observed when trained on uncorrelated trials—simulation results are shown in Fig 11. Also this prediction fits with our experimental data shown in Fig 8.

## Discussion

In this study, we designed a three-dimensional reaching task where we could displace the visual feedback of participants’ hand positions with a two-dimensional translational shift. The statistics over the shift could be learnt by participants in training trials with precise visual feedback. We imposed a correlation between the two dimensions of the shift as a statistical structural invariant and found that participants gradually learnt this structural invariant during training. Participants exploited the structural knowledge to facilitate sensorimotor integration in test trials with partial feedback where the visual feedback was completely uninformative in one dimension. However, we only found this to be the case when participants had binary reward-feedback at the end of these trials. We also recorded a control group, where the correlation structure was absent during training but could have potentially been learnt through the binary reward-feedback in test trials. We found no statistically significant evidence that the correlation structure was learnt over the course of the experiment in the control group. We also found that explicit instructions about the nature of the perturbation and the optimal compensatory response did not enhance participants’ performance, which suggests that they were not following a cognitive strategy. In all groups, we used trials without any visual feedback to probe participants’ prior beliefs over the shift and found that participants in all groups rapidly learnt the mean shift. Our results show that participants in our experiment were indeed able to extract structural invariants in order to enhance their performance in a Bayesian sensorimotor integration task.

In our experiment participants never learnt the correlation structure perfectly. A perfect Bayesian actor with full knowledge of the correlation should show the same behavior in partial feedback trials as in full feedback trials, as one fully visible dimension with correlation structure contains in principle the same amount of information as two fully visible dimensions. This raises the question whether learning of the correlation was still ongoing after four days of training or whether learning of the correlation is imperfect. In the latter case our results would hint at sub-optimal behavior in Bayesian integration tasks. The results in Fig 6A and 6C showing the learning progress over the course of the experiment suggest that learning had not yet flattened out by the end of the fourth session and participants would potentially continue to improve their performance in subsequent sessions of the experiment. Therefore, we cannot distinguish between these two possibilities in our data.

In control experiments we found that reward-feedback was crucial in order to improve the response in partial feedback trials. This raises the question why the group without binary reward-feedback would fail to show improvements under partial feedback despite undergoing training with correlated full feedback trials? There are at least three possibilities. First, this group might have lacked incentive in partial and no-feedback trials, as there was no performance feedback and therefore they might not have cared about their action. Second, this group might have not been able to transfer their skill from full feedback to partial feedback without additional reward cues, as the stimuli in the full- and partial-feedback conditions looked different. Third, this group might have failed to learn the correlation altogether, as in full feedback trials knowledge of the correlation is not necessary to perform well. The third hypothesis is unlikely as in previous studies of sensorimotor integration in a single dimension [8] participants were shown to learn the Bayesian prior despite the absence of any reward feedback in partial and no feedback conditions. While we cannot distinguish between the first two possibilities, our results seem to suggest that learning of the correlation in the full-feedback trials would narrow down the hypothesis space regarding the shifts sufficiently such that participants could exploit the reward-feedback either simply as an incentive (first possibility) or as a reinforcement learning signal for efficient adaptation (possibility two). In any case, the results of the uncorrelated group show that reinforcement learning with binary reward-feedback by itself is not sufficient to learn the correlation structure.

In no-feedback trials we found that participants of the correlated group showed a correlation between the vertical and horizontal dimension of their final hand position. This cannot be explained by a perfect Bayesian actor model that simply compensates the mean shift in no-feedback trials. The two possibilities we considered that could explain this finding are first, trial-by-trial adaptation of participants and second, a sampling strategy where participants sample beliefs from the prior distribution and act accordingly. In the first case, the correlation in no-feedback trials would simply show up in the correlated group as an aftereffect of the previous correlated full feedback trial. In the second case, participants would behave as bounded optimal Bayesian actors that actively sample from the learnt prior distribution rather than just picking the maximum [39–45]. Since the prior distribution exhibits the correlation structure, such a bounded optimal actor would also reflect the correlation structure in their actions in no-feedback trials. We found evidence for both hypotheses and, indeed, they are not mutually exclusive, as a bounded rational actor could be implemented by a Monte-Carlo sampler that naturally introduces trial-by-trial correlations, because all changes in strategy are always stepwise [46, 47].

The sensorimotor integration of different sources of information has been studied previously, in particular the combination of information from different sensory modalities with different reliability and the combination of prior experience with feedback information [7–12, 19, 20]. Other studies have investigated how motor behavior adapts when perturbation statistics change dynamically across trials [21, 23, 24]. Our task belongs to the first category of studies, as there is no trial-by-trial dynamics of the perturbation, just samples from a stationary distribution. Most of these previous studies have reported a quantitative agreement between their data and Bayesian model predictions. Our task is an extension of [8], where the authors used a two-dimensional reaching task with a one-dimensional visuomotor shift to show that the human sensorimotor system optimally combines prior expectations of a hidden variable with noisy visual feedback. In their task, feedback of the virtual hand position was provided by isotropic Gaussian point clouds. In extension of this previous work, we investigate the role of higher-level statistical structure during Bayesian sensorimotor integration. The three-dimensional task setup allowed us to impose such higher-level structure in the space of the two-dimensional hidden variable, which was not possible in the planar task design in [8].

Structure learning has been proposed in the literature as an important meta-learning concept for extracting higher-level invariants in behavioral experiments, both in cognitive tasks [22, 48–52] as well as sensorimotor tasks [27, 28, 34, 35, 37, 53, 54]. In this study we investigate how structural invariants in a two-dimensional hidden variable influence sensorimotor integration, that is the combination of prior experience with uncertain feedback, where the feedback uncertainty could be manipulated experimentally. In contrast, previous studies on structure learning in sensorimotor control typically did not manipulate feedback reliability, and studies on Bayesian sensorimotor integration have typically not investigated multi-dimensional hidden variables with structured spaces. In particular, we designed visual feedback conditions where knowledge of the correlation structure allowed the integration of information across the two dimensions of the hidden shift variable. Therefore, in the current experiment the two structures (correlated vs. uncorrelated) can be subsumed by a single model with parameter *ρ* and the structure learning problem can be cast as learning the prior over *ρ* (or the covariance matrix). However, in general it need not always be the case that the models are nested. In the nomenclature of Bayesian networks structure learning refers in general to learning the dependencies between multiple (hidden) variables. These dependencies can be represented by multiple model classes *M*, such that structure learning implies learning a prior *p*(*M*) over the model classes *M*. Upon arrival of new evidence, the sensorimotor system can then decide between the different models—see for example [37, 53, 54].

In our current paper, our results demonstrate that participants who were trained on a correlation structure could use their structural knowledge to guide their adaptation in test trials with binary reward-feedback. In contrast, participants in the control group that were not exposed to the correlation structure during training were unable to learn the structure in test trials from binary feedback. In summary, we find that structural invariants of hidden variables play an important role in the sensorimotor integration process of combining sensory feedback with prior experience. We find this process to be consistent with Bayesian inference.

## Materials and Methods

### Ethics statement

The study was approved by the ethics committee of the Max Planck Society (reference number: 0269/2010BO2). All participants gave written informed consent.

### Participants

Sixteen female and eight male participants were recruited from the student population of the University of Tübingen. All participants were naive and the local standard rate of eight Euros per hour was paid for participation in the study.

### Materials

We used a virtual reality setup consisting of a Sensable^®^ Phantom^®^ Premium 1.5 High Force manipulandum for tracking participants’ hand movements in three dimensions and an NVIS^®^ nVisor ST50 head-mounted display (HMD) for creating stereoscopic 3D virtual reality. Movement position and velocity were recorded with a rate of 1kHz.

### Experimental design: overview

We designed a 3D-visuomotor task in virtual reality where participants had to perform reaching movements to a fixed target. The participants’ hand position *p*_*h*_ was represented by a shifted hand position *p*_*s*_. In each trial the virtual position *p*_*s*_ was translated in the vertical plane by adding a two-dimensional Gaussian random vector *s* = [*s*_*h*_,*s*_*v*_], such that

$$p_{s}=p_{h}+\;[\begin{array}{c} s_{h}\\ s_{v}\\ 0 \end{array}],$$

where the *z*-dimension corresponds to the veridical forward-backward movement direction and the vertical plane is spanned by *h* (right-left movement direction) and *v* (up-down movement direction). Crucially, the virtual position was only briefly displayed about halfway through the movement, which allowed inference of the unobserved shift depending on the preciseness of the display. As the hidden shift variable was bivariate and Gaussian with *s* ∼ 𝓝(*s*; *μ*, Σ), we could introduce statistical structure between the two dimensions of the shift by correlation, such that

$$\mu =[\begin{array}{c} \mu_{1}\\ \mu_{2} \end{array}]=[\begin{array}{c} -1\\ +1 \end{array}]cm\;\;\;\text{and}\;\;\;\Sigma =[\begin{array}{c} \sigma_{1}^{2}\;\;\;\rho \sigma_{1}\sigma_{2}\\ \rho \sigma_{1}\sigma_{2}\;\;\;\sigma_{2}^{2} \end{array}],$$

with *σ*_1_ = *σ*_2_ = 1*cm* and the correlation coefficient *ρ* depending on the experimental condition. We choose a non-zero mean to be able to assess learning not only through correlation, but also through learning of the mean.

In particular, we trained the first six participants on a correlated 2D-Gaussian distribution over the shift (correlated group, *ρ* = −0.999) and the next six participants on an isotropic 2D-Gaussian distribution (uncorrelated group, *ρ* = 0.0). We refer to these training trials as full feedback trials, where the virtual position was displayed with very low uncertainty in both dimensions of the shift. We tested both groups of participants on a statistically identical set of test trials with either partial feedback or no feedback. Importantly, the shift in these test-trials was always drawn from the correlated 2D-Gaussian (*ρ* = −0.999), regardless of the group. Partial feedback trials were very reliable in one dimension but provided no information about the other dimension of the shift—only if the correlation structure had been learnt successfully, reliable feedback in one dimension allows to infer the shift in the other. No-feedback trials allowed us to test participants’ learnt representation of the prior knowledge over the shift. The different feedback types are illustrated in Fig 1.

### Experimental design: workspace

The workspace of the experiment was ±5*cm* in the left-right direction (*h*-axis), ±5*cm* in the up-down direction (*v*-axis) and 0–14*cm* in the forward-backward direction (*z*-axis). The *h*-*v* plane was tilted by 20° against the vertical direction of gravity to make it approximately perpendicular to participants’ line of sight when looking down at the center of the workspace. The start-position was indicated by a white sphere (radius: 0.6*cm*) centered at (*h*, *v*, *z*) = (0,0,0.5)*cm* and the target was indicated by a yellow sphere (radius: 0.5*cm*) centered at (0,0,14)*cm*. Before initiating the trial by moving into the start sphere, participants’ virtual hand position was veridically displayed by a small cursor (blue sphere, radius: 0.3*cm*). To facilitate 3D-perception, we displayed a grid at the bottom and at the back of the workspace. We also showed a red rectangle moving along the grid to indicate the veridical depth of participants’ virtual hand position.

### Experimental design: trials

To start a trial, participants had to move the cursor representing their hand position into the start-sphere and remain steady for 0.1*s*. After that, a beep indicated the start of the trial. Simultaneously, the start-sphere and the cursor display vanished and the target-sphere was displayed. Participants had a maximum of 2*s* to complete their movement by passing through the target-plane located at 14*cm* in the z-direction—otherwise the trial was repeated. The average trial duration across all participants and trials was 1.041*s*.

After participants had moved 6*cm* into the forward direction towards the target, visual feedback was presented for 150*ms*. The feedback display was dynamic, that is tracking participants hand movements for the duration of the display. There were four different types of visual feedback. In *full feedback* trials (compare Fig 1A), the visual feedback consisted of a small red sphere (radius: 0.3*cm*), centered at the virtual hand position *p*_*s*_. In *partial *s*_*h*_-feedback* trials (compare Fig 1B), participants saw a vertically elongated rectangle centered on the horizontal component of *p*_*s*_ that consisted of 50 small red circles (radius: 0.1*cm*), each circle located randomly within the area spanned by the rectangle and re-sampled at 60*Hz*—compare Fig 1B. The bar stimulus had a width of 0.6*cm* in the horizontal direction and a height that covered the full vertical workspace, thus providing no information about the vertical component of *p*_*s*_. In *partial *s*_*v*_-feedback* trials, participants were shown the same kind of bar stimulus, but this time elongated in the horizontal direction with a height of 0.6*cm* in the vertical direction. The stimulus covered the full horizontal workspace, providing no information about the horizontal component of *p*_*s*_. In *no-feedback* trials (compare Fig 1C), no visual feedback was shown to the participant. Accordingly, participants could only rely on their prior experience in these trials.

A trial was completed, once the participant crossed the vertical target-plane at *z* = 14*cm* in the forward direction. This final hand-position in the vertical plane was analyzed in the Results. Regardless of the visual feedback type of the trial, the participant was informed of whether they had hit the target. A target hit was counted whenever the (potentially non-visible) final cursor position (sphere, radius: 0.3*cm*, corresponding to the final *shifted* hand-position) was intersecting with the target-sphere (radius: 0.5*cm*). To indicate a hit, the target sphere changed its color to green and a rewarding sound was played back. To indicate a miss, the target sphere changed its color to red and a deep-pitched buzzing sound was played back. In full feedback trials, the final cursor position was marked on the grid (blue sphere, radius: 0.3*cm*) in the target-plane (at *z* = 14*cm*).

In order to start a new trial, participants had to return their hand position to the start sphere. Since they did not see their shifted hand position represented by the cursor throughout the trial, they could use the highlighted rectangle on the grid to judge the cursor’s depth. Once they moved their hand into the front half of the work space (*z* ≤ 7*cm*), the target-sphere and any additional final feedback (in case of full feedback trials) disappeared. Instead, the start-sphere and the veridical cursor were displayed. Participants were allowed to take breaks whenever they wanted in this inter-trial phase. Importantly, when participants returned to the start position after completion of a trial the cursor was faded out and no visual feedback of their hand position was shown. When getting close to the start position their hand position was shown veridically. Participants would thus not experience an abrupt jump in the cursor when returning to the start-position.

### Experimental design: sessions

For each participant the experiment consisted of four sessions, spread over four days, with each session consisting of 1000 completed trials (see Fig 2). The first session included a three-staged training-phase (with full feedback trials only): for the first 50 trials there was no shift and the veridical cursor was displayed throughout the whole movement. In the subsequent 50 trials the shifted cursor was displayed throughout the whole trial and participants could see the jump from veridical to shifted cursor after movement onset. In the last training stage (the following 100 trials) only full feedback trials were presented, but no cursor was shown during the trial (except for the brief visual feedback of 150*ms* duration). After the training stage the different feedback types were presented in randomly interspersed order with the following probabilities: 1/2 for full feedback trials and 1/6 for partial *s*_*h*_-feedback, partial *s*_*v*_-feedback and no-feedback respectively. The second, third and fourth session did not include a training phase.

### Experimental design: instructions

Participants were informed that their task was to hit the target with the virtual cursor and that the virtual cursor would “jump” immediately after movement onset (as they would experience in the second training stage). They were informed about the different feedback types and were told that in case of partial feedback the virtual cursor was somewhere behind the flickering bar and could not be outside the bar. In no-feedback trials they were instructed to guess where the cursor might have jumped to and try to blindly hit the target. As an additional incentive participants were shown their overall hit-ratio in partial- and no-feedback trials as a percentage above the workspace. Performance in full-feedback trials did not count towards this hit-rate display.

### Experimental design: control experiments

We introduced two control groups (six participants each) to study the influence of explicit performance signals in partial and no-feedback trials and the potential impact of cognitive strategies. Like the correlated group, both control groups were exposed to correlated shifts in all feedback conditions. In the first control group, the *correlated group without auditory feedback*, participants did not receive any performance feedback about whether they had hit the target in partial- and no-feedback trials. This means that in these trials, the target color did not change according to whether the target was hit or not and a neutral sound was played back instead of the sounds indicating a hit or a miss. Additionally the hit-rate percentage in partial- and no-feedback trials was not shown to participants.

The second control group, the *correlated group with instruction* received additional instructions at the beginning of the experiment. In particular, they were informed about the correlation of the horizontal and vertical dimension of the shift. They were instructed as follows: “*If the cursor jumps to the left, it always jumps up as well and if it jumps to the right it always jumps down as well. This also means that if it jumps up it will also jump to the left and if it jumps down it will also jump to the right. This information is particularly useful for the trials with the bar-feedback*”. Participants were reminded of this instruction after the training phase ended in the first session and again before starting the second session. In order to test for trial-by-trial correlations between full feedback and no-feedback trials in this group, in approximately 8% of all trials an uncorrelated shift stimulus was presented in the full feedback trial just before a no-feedback trial. Uncorrelated full feedback trials never preceded a partial feedback trial, which is the trial type we used to evaluate learning of the correlation structure. Importantly, therefore, these uncorrelated trials do not affect the validity of the control experiment, because a cognitive strategy in partial feedback trials should not depend on the statistics of previous trials, especially if they do not directly precede.

### Computational model: Bayesian sensorimotor integration

The visual feedback *d* = [*d_h_*, *d_v_*]^T^ is modeled using a Gaussian likelihood model: *p*(*d*∣*s*) = 𝓝(*d*;*s*,Σ_obs_). The off-diagonal entries of Σ_obs_ are zero, whereas the diagonal entries depend on the visual feedback type of the trial, that is

$$\Sigma_{\text{obs}}=[\begin{array}{c} \sigma_{h}^{2}\;\;\;\;\;0\\ 0\;\;\;\;\;\sigma_{v}^{2} \end{array}],$$

In the full feedback trials both, the variance in *h*- and *v*-dimension are very low, in no-feedback trials the variance in both dimensions is infinite and in partial feedback trials the variance in one dimension is low whereas it is infinite in the other dimension. The posterior-belief over the shift *s* given the visual feedback *d* is obtained by combining prior knowledge over the shift with the likelihood model—leading to a Bayesian integration of both sources of information:

$$p\left(s|d\right)=\frac{p(d|s)p(s)}{\int p(d|s)p(s)ds},$$ (1)

where the likelihood model is *p*(*d*∣*s*) = 𝓝(*d*; *s*, Σ_obs_) and the prior is given by *p*(*s*) = 𝓝(*s*; *μ*, Σ) as described in Experimental design: overview.

If both the prior and the likelihood are Gaussian, the posterior can also be expressed as a Gaussian distribution *p*(*s*∣*d*) = 𝓝(*s*; *μ*_*p*_, Σ_*p*_)

$$\mu_{p}=\Sigma_{p}(\Sigma_{\text{obs}}^{-1}d+\Sigma^{-1}\mu )$$ (2)

$$\Sigma_{p}={\left(\Sigma^{-1}+\Sigma_{\text{obs}}^{-1}\right)}^{-1}$$ (3)

with mean *μ*_*p*_ and covariance Σ_*p*_. The parameters *μ* and Σ denote the mean and covariance-matrix of the prior and correspond to the parameters of the true distribution over the shift

$$\mu =[\begin{array}{c} \mu_{1}\\ \mu_{2} \end{array}]\;\;\;\text{and}\;\;\;\Sigma =[\begin{array}{c} \sigma_{1}^{2}\;\;\;\rho \sigma_{1}\sigma_{2}\\ \rho \sigma_{1}\sigma_{2}\;\;\;\sigma_{2}^{2} \end{array}],$$

In the four feedback conditions of our experiment, Eq (2) simplifies further to

- Full feedback condition (*σ*_*h*_ → 0 and *σ*_*v*_ → 0)
  $$\mu_{p}=[\begin{array}{c} d_{h}\\ d_{v} \end{array}]=d$$
- Partial *s*_*h*_-feedback condition (*σ*_*v*_ → ∞)
  $$\mu_{p}=[\begin{array}{c} \;\frac{\sigma_{1}^{2}}{\sigma_{h}^{2}+\sigma_{1}^{2}}\;\;\;\;\;\;0\\ \rho \frac{\sigma_{1}\sigma_{2}}{\sigma_{h}^{2}+\sigma_{1}^{2}}\;\;\;\;\;0 \end{array}]\;[\begin{array}{c} d_{h}\\ d_{v} \end{array}]+[\begin{array}{c} \;\;\;\;\frac{\sigma_{h}^{2}}{\sigma_{h}^{2}+\sigma_{1}^{2}}\;\;\;\;\;\;0\\ -\rho \frac{\sigma_{1}\sigma_{2}}{\sigma_{h}^{2}+\sigma_{1}^{2}}\;\;\;\;\;1 \end{array}]\;[\begin{array}{c} \mu_{1}\\ \mu_{2} \end{array}]$$
- Partial *s*_*v*_-feedback condition (*σ*_*h*_ → ∞)
  $$\mu_{p}=[\begin{array}{c} 0\;\;\;\;\;\rho \frac{\sigma_{1}\sigma_{2}}{\sigma_{v}^{2}+\sigma_{2}^{2}}\\ 0\;\;\;\;\;\;\frac{\sigma_{2}^{2}}{\sigma_{v}^{2}+\sigma_{2}^{2}}\; \end{array}]\;[\begin{array}{c} d_{h}\\ d_{v} \end{array}]+[\begin{array}{c} 1\;\;\;-\rho \frac{\sigma_{1}\sigma_{2}}{\sigma_{v}^{2}+\sigma_{2}^{2}}\\ 0\;\;\;\;\;\;\;\;\;\frac{\sigma_{v}^{2}}{\sigma_{v}^{2}+\sigma_{2}^{2}}\; \end{array}]\;[\begin{array}{c} \mu_{1}\\ \mu_{2} \end{array}]$$
- no-feedback condition (*σ*_*h*_ → ∞ and *σ*_*v*_ → ∞)
  $$\mu_{p}=[\begin{array}{c} \mu_{h}\\ \mu_{v} \end{array}]=\mu$$

If participants maximized their hitting chances by following the maximum of the posterior given by *μ*_*p*_, the only difference between the correlated and uncorrelated group occurs in the partial feedback conditions. In the uncorrelated group with *ρ* = 0, participants would integrate the informative feedback dimension with their prior information about this dimension, and they would solely rely on the prior in the uninformative feedback dimension. In the correlated group with *ρ* = −0.999 participants would differ from the uncorrelated group in how they process the uninformative feedback dimension by generating an estimate of the uninformative feedback dimension that relies on the informative feedback dimension and prior expectations on both dimensions.

### Computational Model: hierarchical learning of correlation structure

In the previous section, the Bayesian integration of visual feedback information and prior knowledge about the shift requires knowledge about the parameters *μ*, Σ of the prior over the shift *p*(*s*) = 𝓝(*s*; *μ*, Σ). In our experiment however, these parameters must be learnt by participants over the course of the experiment. In the Bayesian framework this learning process can be modeled by assuming a prior distribution over these parameters—the so-called hyper-prior—and updating the hyper-prior distribution in light of new observations in a Bayesian fashion. In our case the hyper-prior is again a parametric distribution (a normal inverse-Wishart distribution), which allows for a sequential Bayesian update of the parameters of this distribution, sometimes referred to as hyper-parameters. In the following model, the hyper-parameters are updated through the observed shifts in training-trials, that is in full feedback trials, only.

We denote the previously observed shifts in full feedback trials by 𝓓 = {*d*_1_, …, *d*_*N*_}. Ultimately, we seek the belief over the shift *s* in the current trial after observing the visual feedback *d* and after having observed the previous training trials 𝓓. This belief is formalized as the distribution *p*(*s*∣*d*,𝓓). While an optimal Bayesian actor would respond with an action that corresponds to the (negative) mode of this belief, a bounded-rational Bayesian actor would sample beliefs from the distribution *p*(*s*∣*d*,𝓓) and base its movement response on these samples. In our case, we draw a single sample $\tilde{s}∼p(s|d,𝓓)$ and respond with $\tilde{r}=-\tilde{s}$. The distribution *p*(*s*∣*d*,𝓓) is given by Bayes’ rule

$$p\left(s|d,𝓓\right)=\frac{p(d|s)p(s|𝓓)}{p(d|𝓓)}.$$

The Gaussian likelihood model *p*(*d*∣*s*) = 𝓝(*d*; *s*, Σ_obs_) remains the same as in the previous section. Additionally, we have introduced a data-dependent prior *p*(*s*∣𝓓) that models the prior belief about the shift *s* after having observed the training data 𝓓. The prior over the shift *p*(*s*∣𝓓) depends on the update of the hyper-parameters *μ*_0_, Σ_0_ that specify the distribution *p*(*s*∣*μ*_0_, Σ_0_). The update of the hyper-parameters is modeled probabilistically through *p*(*μ*_0_, Σ_0_∣𝓓, Σ_obs_). This allows us to specify a model for Bayesian integration of prior beliefs and feedback information, where the prior beliefs are data-dependent:

$$p\left(s|d,𝓓\right)=\frac{p(d|s)p(s|𝓓)}{p(d|𝓓)}=\frac{p\left(d|s\right)\int d\mu_{0}d\Sigma_{0}\;p\left(s|\mu_{0},\Sigma_{0}\right)p\left(\mu_{0},\Sigma_{0}|𝓓,\Sigma_{\text{obs}}\right)}{p(d|𝓓)}.$$ (4)

where the update equation for the hyper-parameters *μ*_0_, Σ_0_ is given by

$$p\left(\mu_{0},\Sigma_{0}|𝓓,\Sigma_{\text{obs}}\right)=\frac{p\left(𝓓|\mu_{0},\Sigma_{0},\Sigma_{\text{obs}}\right)p\left(\mu_{0},\Sigma_{0}\right)}{p(𝓓|\Sigma_{\text{obs}})},$$ (5)

with

$$\begin{array}{ccc} p(𝓓|\mu_{0},\Sigma_{0},\Sigma_{\text{obs}}) & = & \prod_{i=1}^{N}p\left(d_{i}|\mu_{0},\Sigma_{0},\Sigma_{\text{obs}}\right)\\ & = & \prod_{i=1}^{N}\int ds\;\underset{N(d_{i};s,\Sigma_{\text{obs}})}{\underset{︸}{p(d_{i}|s,\Sigma_{\text{obs}})}}\;\underset{N(s;\mu_{0},\Sigma_{0})}{\underset{︸}{p(s|\mu_{0},\Sigma_{0})}}\\ & = & \prod_{i=1}^{N}N\left(d_{i};\mu_{0},\Sigma_{0}+\Sigma_{\text{obs}}\right)=\prod_{i=1}^{N}N\left(d_{i};\psi ,\Theta \right). \end{array}$$ (6)

It is crucial to note that the likelihood of a previously observed data point *d*_*i*_ has a Gaussian form 𝓝(*d*_*i*_; *μ*_0_, Σ_0_+Σ_obs_) = 𝓝(*d*_*i*_; *ψ*, Θ)—see the standard textbooks [55, 56] by Bishop (2.115) or Murphy (4.126). If we replace *μ*_0_,Σ_0_ with *ψ*,Θ in Eq (5) and subsume Σ_obs_, we can use a *normal inverse-Wishart* distribution as a prior distribution *p*(*ψ*,Θ) = NIW(*ψ*,Θ), which is the conjugate prior for a Gaussian with unknown mean and covariance matrix. Conveniently, this leads to closed-form sequential update equations for the posterior parameters of the normal inverse-Wishart distribution after having observed *N* data-points.

$$p\left(\psi ,\Theta |{𝓓}_{N}\right)=\frac{p\left({𝓓}_{N}|\psi ,\Theta \right)p\left(\psi ,\Theta \right)}{p({𝓓}_{N})}=\text{NIW}\left(\psi ,\Theta |m_{N},\kappa_{N},\nu_{N},S_{N}\right)$$ (7)

$$m_{N}=\frac{\kappa_{0}m_{0}+N\bar{D}}{\kappa_{N}}$$ (8)

$$\kappa_{N}=\kappa_{0}+N$$ (9)

$$\nu_{N}=\nu_{0}+N$$ (10)

$$S_{N}=S_{0}+S_{\bar{D}}+\frac{\kappa_{0}N}{\kappa_{0}+N}\left(\bar{D}-m_{0}\right){\left(\bar{D}-m_{0}\right)}^{T}$$ (11)

with $\bar{D}=\frac{1}{N}\sum_{i=1}^{N}d_{i}$ being the empirical mean-shift and $S_{\bar{D}}={\sum_{i=1}^{N}\left(d_{i}-\bar{D}\right)\left(d_{i}-\bar{D}\right)}^{\text{T}}$ (see [56] Murphy section 4.6.3.3).

Putting it all together (and correcting for the subsumed Σ_obs_ in the hyper-prior) we get the following rejection-sampling scheme to simulate a participant:

1. Sample from *p*(*μ*_0_,Σ_0_∣𝓓,Σ_obs_) as given by Eq (5)
  - Draw a sample from the normal inverse-Wishart $\tilde{\psi },\tilde{\Theta }∼p(\psi ,\Theta |{𝓓}_{N})$
  - ${\tilde{\mu }}_{0}=\tilde{\psi }$ (follows from the last equality in Eq (6))
  - ${\tilde{\Sigma }}_{0}=\tilde{\Theta }-\Sigma_{\text{obs}}$ (follows from the last equality in Eq (6), always use the full feedback Σ_obs_ in this particular step as the model is trained on full feedback trials only)
  - If ${\tilde{\Sigma }}_{0}$ is not positive semi-definite (that is if it has eigenvalues ≤ 0), discard samples and re-start at the first step, otherwise continue.
2. For a given ${\tilde{\mu }}_{0},{\tilde{\Sigma }}_{0}$, draw a sample from $\tilde{s}∼p(s|{\tilde{\mu }}_{0},{\tilde{\Sigma }}_{0})$.
3. Perform a rejection-acceptance step with the likelihood of the observed feedback given the sampled shift $p(d|\tilde{s})$. To do so evaluate if $u\leq p(d|\tilde{s})/l_{\text{max}}$ for *u* ∼ 𝓤[0; 1] and accept the sample if the inequality holds or reject otherwise. *l*_max_ is the maximum value of the likelihood given by $l_{\text{max}}=1/\sqrt{|\Sigma_{\text{obs}}|{\left(2\pi \right)}^{2}}$, where ∣ ⋅ ∣ denotes the determinant.
4. If the sample was accepted, respond to the stimulus with a response $\tilde{r}=-\tilde{s}$. If the sample was rejected, restart at the first step.
5. In case of a full feedback trial, update the parameters of the normal inverse-Wishart with the sequential update rules for the parameters following Eqs (8)–(11).

For our simulation we used the following parameters. The initial belief about the mean-shift was chosen as *m*_0_ = [0, 0]^T^ with an initial weight of *κ*_0_ = 300. The initial belief about the covariance matrix was set to a diagonal matrix (no correlation between the horizontal and vertical dimension) with a variance of one for both dimensions with an initial weight of *ν*_0_ = 3000. For the inverse-Wishart prior, *S*_0_ must then be specified in the following way:

$$S_{0}=[\begin{array}{c} \nu_{0}\;\;\;\;0\\ 0\;\;\;\;\nu_{0} \end{array}].$$

The weights of the initial beliefs *κ*_0_ and *ν*_0_ were determined by averaging over 30 simulation runs and then comparing the resulting medians of the quantities shown in Fig 10 and Fig 11 to the medians obtained from the participants of the correlated group (that is the median slopes in horizontal and vertical dimension, the median means in both dimensions as well as the median correlation in no feedback trials). In particular, we performed a grid-search over a range of parameter-values such that the sum-of-squared-errors between the time course of simulated medians and the participants’ median was minimized. We found that the weights on the initial beliefs directly govern the learning-rates (as expected), which allows to reproduce a broad range of learning-behavior. The results obtained are not particularly sensitive to small changes in the parameters.

The results shown in Fig 10 and Fig 11 were obtained by taking the median over six virtual participants with the best-fit parameters. In the figure we compare two different runs—one run where the model was trained on correlated shifts (identical to the shifts experienced by the six participants in the correlated group) and another run where the model was trained on uncorrelated shifts (identical to the shifts experienced by the six participants of the uncorrelated group) without changing the model parameters.

The covariance matrix of the observation noise Σ_obs_ was dependent on the trial type, but was always a diagonal matrix (no correlation in the observation noise). For full feedback trials, both diagonal entries were set to 0.2*cm* ^2^ reflecting reliable feedback. For partial-*s*_*h*_ feedback trials the entry for the horizontal dimension was 0.2*cm* ^2^ and the entry for the vertical dimensions was set to 40*cm* ^2^ as the feedback provided reliable information in the horizontal dimension and no information in the vertical dimension. For the partial *s*_*v*_ feedback trials the entries were reversed—the horizontal dimension was set to 40*cm* ^2^ and the entry for the vertical dimensions was 0.2*cm* ^2^. For the no feedback trials both diagonal entries were set to 40*cm* ^2^ as the feedback provided no information about the shift in either dimension.

## Supporting Information

S1 Fig Evolution of slopes in partial feedback trials—individual participants and group medians for correlated group without auditory feedback and correlated group with instruction. Changes in slope in partial feedback trials. The slope is a performance measure determined as in Fig 3 in the main manuscript but using a sliding window of 100 trials. For the analysis only partial *s*_*v*_- or partial *s*_*h*_-feedback trials were taken out from the pooled data across all sessions. Thin colored lines indicate individual participants and can vary in length since the exact number of relevant trials could fluctuate due to the probabilistic generation of trials. The thick black line shows the median over participants—taking only into account trials where data from all participants exists. The marked ticks on the x-axis at the bottom of the figure indicate the end of the corresponding session (on average). **A** Evolution of the horizontal slopes in partial *s*_*v*_ feedback trials of the correlated group without auditory feedback. Horizontal information is not given by the feedback, but can only be obtained through knowledge of the correlation structure. **B** Same as A but showing data of the instructed group. **C** Evolution of the vertical slopes in *s*_*h*_ feedback trials of the correlated group without auditory feedback. Vertical information is not given by the feedback, but can only be obtained through knowledge of the correlation structure. **D** Same as C but showing data of the instructed group.
(EPS)

S2 Fig Evolution of means in no feedback trials—individual participants and group medians for correlated group without auditory feedback and correlated group with instruction. Learning of mean shift over all sessions revealed by performance in no-feedback trials averaged over a sliding window of 100 trials. For the analysis only no-feedback trials were taken out from the pooled data across all sessions. Thin colored lines indicate individual participants and can vary in length since the exact number of relevant trials could fluctuate due to the probabilistic generation of trials. Thick black lines show the median over participants—taking only into account trials where data from all participants exists. The bar at the bottom of the figure indicates the corresponding session (on average). **A** Learning of the mean in the horizontal dimension of the correlated group without auditory feedback. **B** Same as A but showing data of the instructed group. **C** Learning of the mean in the vertical dimension of the correlated group without auditory feedback. **D** Same as C but showing data of the instructed group. **E** Learning of the mean in the horizontal dimension—showing the medians of all groups of participants. **F** Learning of the mean in the vertical dimension—showing the medians of all groups of participants.
(EPS)

S3 Fig Evolution of xy-correlation in partial feedback trials and low-uncertainty-dimension slopes in partial feedback trials—medians for all groups. Changes in correlation and low-uncertainty-slope in partial feedback trials using a sliding window of 100 trials. For the analysis only partial *s*_*v*_- or partial *s*_*h*_-feedback trials were taken out from the pooled data across all sessions. Different colored lines show the median over the different groups of participants and can vary in length since the exact number of relevant trials could fluctuate due to the probabilistic generation of trials. The marked ticks on the x-axis at the bottom of the figure indicate the end of the corresponding session (on average) **A** Adaptation of correlation between the vertical and horizontal terminal hand position measured in partial *s*_*h*_-feedback trials. Large magnitudes of the correlation indicate a more “diagonal” movement, which is required by the optimal response in these trials. **B** Adaptation of correlation between the vertical and horizontal terminal hand position measured in partial *s*_*v*_-feedback trials. Large magnitudes of the correlation indicate a more “diagonal” movement, which is required by the optimal response in these trials. **C** Evolution of the horizontal slopes in partial *s*_*h*_ feedback trials where horizontal information is given by the feedback with low uncertainty. Ideally, the value of this slope would be close to zero. **D** Evolution of the vertical slopes in partial *s*_*v*_ feedback trials where vertical information is given by the feedback with low uncertainty. Ideally, the value of this slope would be close to zero. In the upper panels it can be seen that the instructed group initially shows an increased magnitude in movement correlation in partial-feedback trials which indicates that they understood and followed the instruction. However in Fig 9 in the main manuscript it can be seen that the instructed group does not have a decreased slope in these trials. In contrast, their slope in the low-uncertainty dimension in these trials was increased compared to the other groups (shown in lower panels of this figure). This suggests that the instruction was not helpful but rather impeded their shift-compensation in the low-uncertainty dimension.
(EPS)

S1 Dataset Data recorded from the experiment. All data required for reproducing the results and figures presented in the paper.
(ZIP)
